# Exogenous Fecal Microbial Transplantation Alters Fearfulness, Intestinal Morphology, and Gut Microbiota in Broilers

**DOI:** 10.3389/fvets.2021.706987

**Published:** 2021-10-01

**Authors:** Chao Yan, Jinlong Xiao, Zhiwei Li, Hao Liu, Xinjie Zhao, Jian Liu, Siyu Chen, Xingbo Zhao

**Affiliations:** ^1^College of Animal Science and Technology, China Agricultural University, Beijing, China; ^2^Guizhou Nayong Professor Workstation of China Agricultural University, Bijie, China; ^3^Guangdong Provincial Key Laboratory of Animal Molecular Design and Precise Breeding, Key Laboratory of Animal Molecular Design and Precise Breeding of Guangdong Higher Education Institutes, School of Life Science and Engineering, Foshan University, Foshan, China

**Keywords:** fecal microbiota transplantation, gut microbiota, fearfulness, intestine, broiler

## Abstract

Fecal microbiota transplantation (FMT) documented transplanting a donor fecal sample to a receipt individual for a desired physiologic effect. However, whether the gut microbiota construction, intestinal maturation, and behavioral plasticity are modulated by FMT during the early life of broilers is waiting for verification. To evaluate the role of transfer of fecal microbiota from aged broilers donor (BD) to another individual, 96 birds were equally divided into a check (CK, control) group and a broiler recipient (BR) group. FMT was conducted daily from 5 to 12 days of age to determine the future impact on body weight, behavior, intestinal development, and gut microbiota. Results indicated that fearfulness in the CK group was higher than the BR group in both the behavioral tests (*p* < 0.05). The muscularis mucosa, thickness of muscle layer, and thickness of serous membrane layer in the BR group were higher compared with those of the CK group in the jejunum (*p* < 0.05). In the gut microbiota, Shannon diversity showed no difference, while beta diversity presented a difference in principal coordination analysis (PCoA) between the CK and BR groups. At the phylum level, the relative abundance of *Lentisphaerae* in the CK group was lower than the BR (*p* = 0.052) and BD (*p* = 0.054) groups. The relative abundance of *Tenericutes* in the BD group was higher than that in the CK and BR groups (*p* < 0.05). At the genus level, *Megamonas* in the CK group was higher than the BR (*p* = 0.06) and BD (*p* < 0.05) groups. In the BR group, the functional capabilities of microbial communities analyzed by the Kyoto Encyclopedia of Genes and Genomes (KEGG) pathway were increased in the glutamatergic synapse and N-glycan biosynthesis pathways in comparison with the CK and BD groups (*p* < 0.05). Some characteristics of gut microbiota in the donor chickens could be transferred to recipient chickens by FMT. In conclusion, exogenous FMT as a probiotic-like administration might be an efficient way to improve the physiology and behavior of chickens. Notably, the role of microbiota for various individuals and periods remains undefined, and the mechanism of microbiota on behaviors still needs further investigation.

## Introduction

Gut microbiota is closely associated with broad consequences for enteric health, diseases, and performance (1, 2), whose sensitivity and maturity coincide with early life development (1, 3). Early-life stage is the vital period for the establishment of intestinal microbiota, and development (4, 5), contributing to health outcomes in later life of animals (5, 6). The critical effects can be attributed to the increasing of the beneficial bacteria, and regulation of metabolites on the intestinal epithelial barrier, production, hormones, and immune system (1, 2). Although the intestinal system of chicks was anatomically completed in the embryonic stage, the size, morphology, and mucosal enzyme activity of small intestines in chicken were determined from hatching to 12 days of age (7). As is known to all, the development and maturation of intestinal morphology and function are vital for individual growth and development (8). Therefore, modulating post-hatch microbial colonization is essential for understanding gut microbiota construction, intestinal maturation, and physiological plasticity during the early life of broilers.

Gut microbiome is influenced by diet, age, rearing system, and the supplement of prebiotics (9–11). Compared with these factors, fecal microbiota transplantation (FMT), serving as a tool, can transfer the fecal material from one individual to another to achieve a desired physiologic effect, such as treating diseases, altering body weight gain, and improving pathogen tolerance, thereby managing the reconstruction of gut microbial composition and function (9). To date, FMT has been widely applied for treatments of diseases, behaviors, and production performance in a broad species including humans, laboratory and farm animals (10, 12). Germ-free mice implanted with the fecal microbiota from individuals with irritable bowel syndrome having diarrhea showed faster gastrointestinal transit, intestinal barrier dysfunction, innate immune activation, and anxiety-like behavior compared with those transplanted feces from healthy individuals (13). Evidence also elucidates that FMT can improve growth performance and play crucial roles in maintaining the intestinal barrier via modulating the microbial composition (14, 15), alleviating the intestinal barrier injury through the reduction of intestinal permeability (16), as well as enhancing intestinal morphology and integrity (17) in piglets. In beef cattle, FMT can transfer phenotypes and develop effective and selective gut microbiota, which improves feed efficiency (18). Besides, transferring microbiota from diseased bovine to mouse induced disorder of the mucosa structure, including necrosis of epithelial cells, increase of the subepithelial space, and structural damage of the villi (19).

FMT affects host health through increasing beneficial microbiota, the competitive exclusion of pathogens, reducing the community production of growth suppression metabolites, and improving energy metabolism (20). Furthermore, the administration of the fecal microbiota from healthy chickens has been applied to transfer colonization resistance against *Salmonella* to newly hatched chickens (21), and fecal microbiota from high feed-efficient donors during the early age of life could improve the feed efficiency of chickens (22). A previous study also signaled that transferring intestinal microbiota of high-yield laying hens had profound effects on the egg production of low-yield laying hens (23). In particular, post-hatching FMT had impacts on behavioral responses and physiological characteristics related to feather pecking (24). In broilers, FMT can assist building a stable microbial community and protecting against pathogen challenges (25). Besides, the administration of *Bacillus amyloliquefaciens* as a probiotic had an influence on reduced agonistic behavior and distress calls in turkeys (26). Similarly, the supplementation with the probiotic *Pediococcus acidilactici* improves memory and decreases the emotional reactivity in the tonic immobility test (27). Particularly, the related study illustrates that cecal microbiota transplantation can affect emotional reactivity in Japanese quails (28). Thus, FMT as an emerging means is conducive to develop health-compatible phenotypes.

Given the abovementioned advantages of FMT, this study was aimed to explore whether body weight, behavior, intestinal structure, and gut microbiota would be modulated by the post-hatch FMT in broilers. The study will provide evidence for further understanding of interactions among microbiota and characteristics of host gut, and illuminating a possible way of manipulating gut health by utilizing gut microbiota as the key target.

## Materials and Methods

### Animals and Treatments

The experimental protocol was approved by the China Agricultural University Laboratory Animal Welfare and Animal Experimental Ethical Inspection Committee (approval number: CAU20180628-6). The experiment was carried out at an organic farm located at Bijie city, Guizhou province, China. The 96 post-hatching healthy commercial broilers were collected from Guangxi Jinling Agriculture and Animal Husbandry Group Co., Ltd. (Nanning, Guangxi, China) and then randomly divided into two groups, respectively, and equally housed in a total of six cages (1.0 × 1.2 × 0.5 m), with 16 chicks in each cage, at a temperature of 32–34°C for the first week and 28–30°C for the second week. Fecal microbiota transplantation (FMT) was conducted in the experimental broiler recipient (BR) group compared with the check (CK) group. The lighting regime was set as 23 h of light exposure and 1 h of dark treatment during hatching, reducing light exposure by 1 h per day for the first 7 days and remaining at 16 h of light exposure and 8 h of dark treatment afterward. When the brooding environment was adapted by chicks, we conducted the FMT from 5 to 12 days (see the *Preparation and inoculation of the* fecal microbiota transplantation section), and then on day 28, each group of broilers was transferred to a new cage (0.19 × 0.30 × 0.40 m). Four chickens were raised in each cage with a coated wire mesh, with a total of 48 chickens in 12 cages. All chicks had *ad libitum* access to water, as well as a starter feeder and grower feeder (corn–soybean meal diets) provided by New Hope Group, Chengdu, Sichuan, China. Novel arena tests and predator tests were conducted at 42 and 49 days, respectively. The body weights of all chicks were measured at 6:00~7:00 in the morning when fasting on days 42, 49, and 56. On day 61, 10 chicks of each group were randomly collected and humanely slaughtered through electrical waterbath stunning. In brief, all chickens were kept in transport boxes and transported to a local commercial poultry slaughter. After resting for half an hour, the feet of birds were fixed into a grounded metal shackle. For stunning, each chicken was immersed in an electrical waterbath up to the base of their wings. The stunning process had a constant 15 V and a sine wave AC at 500 Hz for 10 s. The parameters used as an unconsciousness indicator was a negative corneal reflex (blinking response elicited by touching the cornea). After stunning, neck cutting was immediately conducted during unconsciousness.

### Preparation and Inoculation of the Fecal Microbiota Transplantation

Feces were collected from four healthy and eligible broilers donors (BD) (52 weeks of age, with more positive physiological functions and behaviors), which were housed in a 2.0 × 1.2 × 1.5-m^3^ cage. The birds were adults, and the gut microbiota was quite mature and stable over time. In addition, the birds were of the same age, had identical genetic backgrounds and feed, as well as were being raised in an identical system and environment, and so on. Therefore, the variation of donor microbiota is limited, which may have a limited effect on FMT in this study. According to the standardized donor preparation and identification for FMT, donor broilers that did not have diarrhea and digestive disorders, never treated with medication, and without antibiotics and probiotics for at least 2 months were screened before feces collection (29). The fecal microbiota was pooled from the four donor birds. The cage had a flooring of wire mesh, which was 0.5 m above the ground. A tray was placed under the wire mesh to facilitate excreta collection, which was cleaned, sanitized, and dried every time before being used. The fresh fecal excreta did not include cecal droppings that were immediately collected when dropped, and then placed on ice. In a sterile environment, the white portion of the excreta that mainly comprised of uric acid was removed. Furthermore, a 1:5 (w/v) solution was obtained by fully dissolving collected feces with 5-ml saline (0.2 g/ml) in a 50-ml beaker (30). Eight layers of medical gauze were placed in the conical flask to separate large particulate matter from the solution. Besides, the primary product was repeated following the first time. After that, 5% glucose was added into the final fecal suspension. Finally, the fecal suspension was inoculated within 2 h. The fecal suspension was daily re-prepared before the FMT period. The 1 ml of donor suspension was mixed with RNA later (SR0020, Solarbio, China) stocked at −20°C on a scale of 1:10 every time until DNA extraction. In the end, seven donor suspensions were pooled with an equivalent, forming a single combined and homogenized sample to test donor microbiota (22, 31).

Before the feces were transplanted, chicks had free access to the feed, while the water was deprived for 1 h. On day 5 after hatching (the reason for choosing 5 days of age was that chicks were considered to take time to adapt to the new environment; on the other hand, gut microbiota was immature and unstable), every BR was orally injected with 0.5 ml of fecal liquid through a 1-ml syringe, which was carried out for 7 days continuously. The CK group was injected with 0.5 ml of saline simultaneously. The transplantation was orally conducted at the back of the tongue, and the birds were supervised while swallowing. Then all chicks were inspected and monitored for an external phenotype.

### Behavioral Test

#### Novel Arena Test

Novel arena tests are well-validated behavioral tests performed on individual birds to observe and assess variations in exploration and general vigilance (32). The motivational states of the test are considered to be fear and anxiety, which can be observed as altered vigilance patterns, in response to social isolation and perceived potential danger in the novel open area. On day 42, 20 randomly selected chickens (one or two from 12 replicates) of each treatment were moved to a test arena (2.0 × 2.5 × 1.5 m) enclosed by solid panels. The test was recorded by video for 15 min. A single observer extracted the data from the videos. The occurrence of “vigilance” (appearing alert, with the head looking around above a horizontal plane) and “foraging” (pecking at the ground) was analyzed by a scanning method, and the behavior was recorded as present or absent every 10 s.

#### Vigilance Test

A predator model was used to examine the difference in fearfulness between individual birds (32). At the age of 49 days, 15 randomly selected chickens (one or two from 12 replicates) of each group were individually placed in a 2.0-m L × 2.0-m W × 1.2-m H test arena simultaneously to test response to a predator. Two polyvinyl chloride material hawks (0.11-m L × 0.27-m W × 0.07-m H) were used as predator models to test the fear response. Before testing, the chickens were deprived of food and water from 18:00 on that day (it can cause the hunger of birds and more motivation to stimulate food-seeking behavior). When tested, the regular feed was placed in one corner of the arena, and regular feed with live worms (a highly valued food resource that birds prefer to eat and are more willing to explore and take risks) (33) was placed in the opposite corner where a hawk model (30-cm L × 30-cm W) was placed 50 cm vertically above the feed. Furthermore, the vocalizations of a hawk were played three times (at 5 and 11 min) during the 12-min test. The immediate behavior reactions in “response to predator” were measured for each chick by using a scale from 0 to 3, where 3 represented the highest level of fear as described in the previous study (32). The test was observed and recorded by video.

### Histological Examination of the Intestine

After slaughtering, three small intestines (jejunum) of each group were preserved and fixed by immersion in 10% neutral-buffered formalin for subsequent examination. The histomorphology was determined as described previously (34). Briefly, approximately 5 cm of the middle of the jejunum was fixed, dehydrated, and embedded in paraffin and cut into sections with a thickness of 5 μm for staining with hematoxylin and eosin. The height villus, crypt depth, thickness of muscularis mucosa, thickness of muscle layer, as well as thickness of serous membrane layer were determined by a single experimenter and measured with an Olympus CK 40 microscope (Olympus Optical Company) at × 40 magnification. Each parameter of the 30 portions from five sections were measured, including villus height, crypt depth, muscularis mucosa thickness, muscle layer thickness, as well as serous membrane layer thickness. Six fields in each intestinal section were considered. After measuring the villus height and crypt depth, the ratio of villus height/crypt depth was calculated.

### Gut Microbiota

Microbial diversity and abundance are most evident in the ceca, which permits more substantial microbial fermentation (35, 36). After slaughtering, 10 chicken cecum contents were randomly collected and stored in dry ice to test the microbiota. The cecum microbiota was measured and analyzed by the 16S rDNA. Once the DNA samples were received, a quality test would be done first. The genomic DNA in each sample was quantified by the Thermo NanoDrop 2,000 spectrophotometer and detected by 1% agarose gel electrophoresis. The selected region of 16S rDNA amplification was the V3–V4 regions region using universal primers, forward primer (5**”**−3”): CCTACGGGRSGCAGCAG (341F) and reverse primer (5”−3”): GGACTACVVGGGTATCTAATC (806R), which were individually barcoded. The qualified DNA was used to construct sequencing libraries, which were sequenced on an Illumina HiSeq PE250 platform and merged to tags. The consensus sequence was generated by Pandaseq (37). The tags were clustered into OTU (operational taxonomic unit) with a 97% sequence similarity threshold by scripts of software USEARCH (v7.0.1090).

Based on the OTU abundance, the OTU of each group was listed, and a Venn diagram was drawn by Perl SVG of software R (v3.1.1), after which the common and specific OTU ID were summarized. Then, the relative abundance of taxonomic ranks, alpha diversity, and beta diversity were analyzed through bioinformatics. Alpha diversity was adopted to explore within-group sample diversity (38). In this study, the Shannon index was applied to analyze alpha diversity, which can detect the richness and evenness of gut microbiota. The index represents that a higher value means a higher alpha diversity. Beta diversity was applied for evaluating differences between groups in species complexity, which was done by software QIIME (v1.80). Both Adonis analysis were performed by the package “ade4” of software R (v3.1.1), and the unweighted Unifrac distance matrices were plotted in the principal coordination analysis (PcoA). Adonis analysis is a non-parametric test to detect whether the differences between groups (two or more groups) are significantly greater than the differences within groups, so as to determine the sensibility of group setting, in which R is between (−1,1), and R > 0, indicates significant difference among groups. R < 0 indicates that the difference within the group is greater than the difference among groups. The reliability of statistical analysis is represented by *p*, and *p* < 0.05 indicates statistical significance. PCoA, one of the beta diversity methods, was used to construct a 2-D graph to summarize contributors that are mainly responsible for the differences of OTU composition in different groups. The similarity will be high if two groups are closely presented. For taxa, the relative abundance analysis was performed by the Kruskal–Wallis test and Wilcoxon rank-sum test at the phylum and genus levels, respectively.

The functional capabilities of microbial communities were predicted by the Phylogenetic Investigation of Communities by Reconstruction of Unobserved State (PICRUSt) (39), which was recaptured key findings from the Human Microbiome Project and predicted the abundance of gene families in host-associated and environmental communities. PICRUSt was carried out to identify the significant Kyoto Encyclopedia of Genes and Genomes (KEGG) differences between the functional potentials of the microbial communities. Variations of KEGG functions among the BD, CK, and BR groups were characterized by the linear discriminant analysis (LDA) effect Size (LEfSe). The threshold on the logarithmic score of LDA analysis was set to |LDA score| >2.0, and *p*-values were calculated with the Kruskal–Wallis test for the non-parametric test when the BD, CK, and BR groups were compared. Significance was established at *p* < 0.05.

### Statistical Analysis

Statistical analyses were performed with IBM SPSS Statistics 21. Body weight met the assumptions for parametric analysis after checking normality and homogeneity of variance and transforming when necessary. They were analyzed using a one-way ANOVA. The behavior in the novel arena test used the Mann–Whitney U-test. The relative to an identified distribution (Ridit) analysis was used to assess the fear score in response to the predator test. Data were presented as mean ± standard error (SE). All values with *p* < 0.05 were regarded as statistically significant.

## Results

### Body Weight

**Figure 1:**
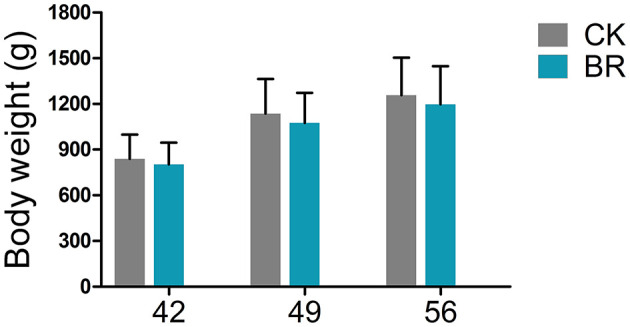
Body weight. CK, check, *n* = 48; BR, broiler recipient, *n* = 48.

The body weight (g) displayed no significant difference between the CK and BR groups on days 42, 49, and 56, respectively (Figure 1).

### Novel Arena Test

**Figure 2:**
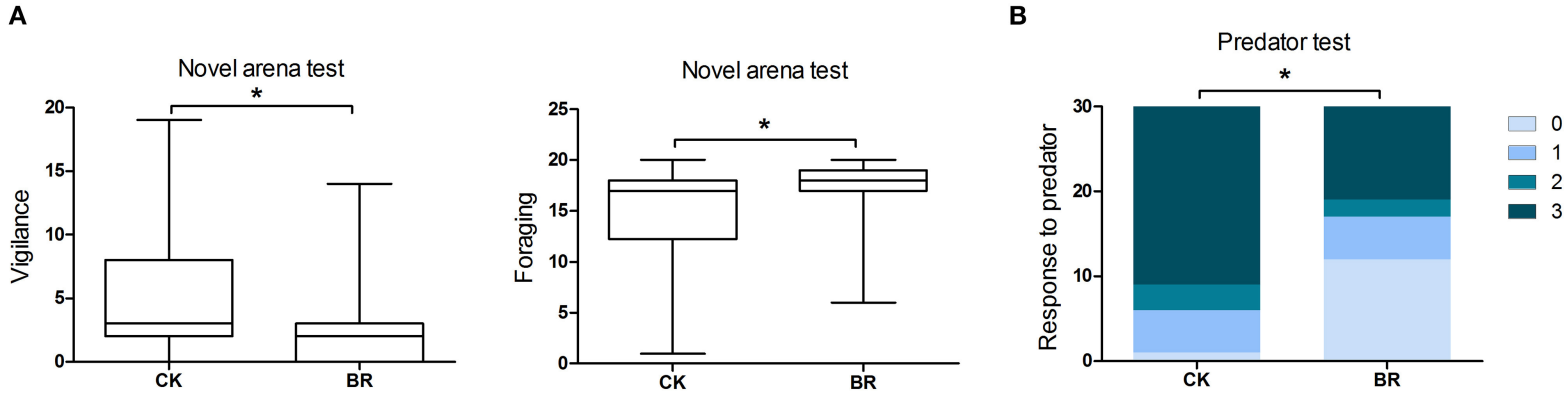
Patterns of behavioral responses of broilers to personality assays. **(A)** Vigilance and foraging (frequency) in the novel arena test; *n* = 20 in each group. **(B)** Response to predators (score 0–3) in the predator test, *n* = 15 in each group. CK, check; BR, broiler recipient.

The foraging in the CK group was lower than that in the BR group (*p* < 0.05, Figure 2A). The vigilance in the CK group was higher than that in the BR group (*p* < 0.05, Figure 2A).

### Vigilance Test

The response to predator in the CK group was higher compared with that of the BR group (*p* < 0.05, Figure 2B).

### Intestinal Morphology Examination

**Figure 3:**
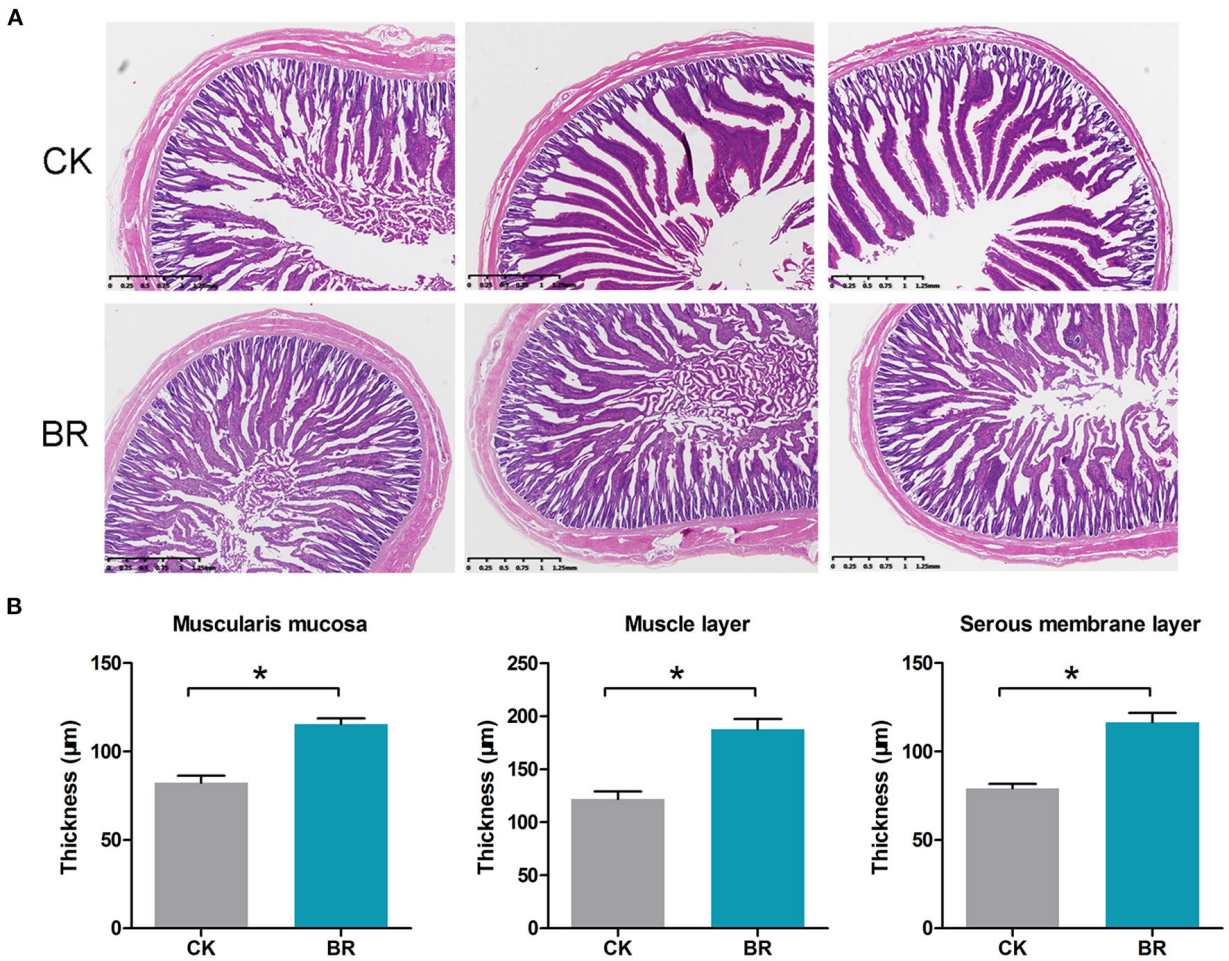
**(A)** The small intestinal tissue histological section. **(B)** Muscularis mucosae thickness, muscle layer thickness, and serous membrane layer thickness were measured (*p* < 0.05). CK, check, *n* = 3; BR, broiler recipient, *n* = 3.

The jejunum morphology (μm) of chickens is shown in Figure 3A. The villus height (1,618.4 ± 75.7 vs. 1,288.0 ± 23.5), crypt depth (338.5 ± 9.8 vs. 410.9 ± 9.1), and the ratio of villus height/crypt depth showed no difference between the CK and BR groups. The muscularis mucosa (115.3 ± 3.3), muscle layer thickness (187.7 ± 9.6) and serous membrane layer thickness (116.5 ± 5.3) in the BR were higher compared with those in the CK group (82.4 ± 3.9, 121.6 ± 7.4, 79.1 ± 2.7) (*p* < 0.05, Figure 3B), accordingly.

### Gut Microbiota

#### Gut Microbiota Composition

**Figure 4:**
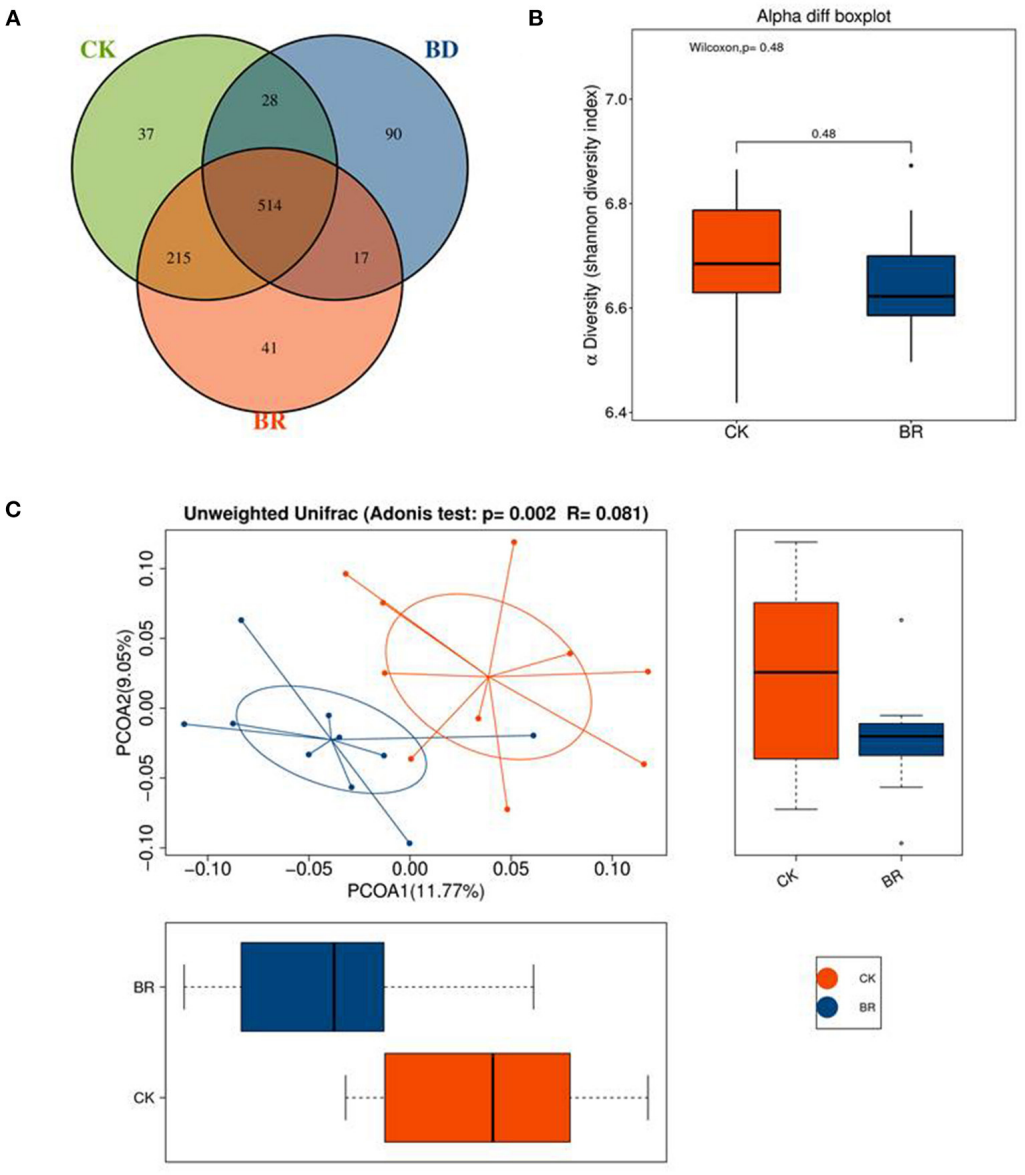
**(A)** The Venn diagram; different color represents different group. The interior of each circle symbolically represents the number of operational taxonomic units (OTUs) in a certain group; the overlapping area represents the set of common OTUs in the counterpart groups; the single-layer zone represents the unique OTUs in a certain group. **(B)** Shannon diversity. **(C)** Principal coordinate analysis (PCoA); the abscissa represents the first principal coordinate, the percentage in brackets represents the contribution rate of the first principal coordinate to the sample difference, the ordinate represents the second principal coordinate, and the percentage in brackets represents the contribution rate of the second principal coordinate to the sample difference. CK, check, *n* = 10; BR, broiler recipient, *n* = 10; BD, broiler donor, a pooled sample of *N* = 1.

A Venn diagram displayed the number of common/unique OTUs across groups (Figure 4A). The three groups had 514 common OTUs. Compared with the CK (*n* = 10) group, the BR (*n* = 10) group and BD (a pooled sample of *N* = 1) group had 729 (514 + 215) and 542 (514 + 28) common OTUs, respectively. Besides, the BD group shared 531 (514 + 17) OTUs with the BR group.

Shannon diversity showed no difference among the CK, BR, and BD groups (Figure 4B). The variation showed a greater dissimilarity among groups than within the CK and BR groups (R = 0.081, *p* < 0.05). Beta diversity was presented in the PCoA plot with different graphic outputs (Figure 4C).

At the phylum level, the top three microbiomes were *Bacteroidetes, Firmicutes*, and *Proteobacteria* among the CK (56.0, 30.27, 4.25%), BR (53.93, 29.57, 4.18%), and BD (36.33, 39.33, 16.17%) groups. However, the relative abundance of *Firmicutes* was higher than *Bacteroidetes* in the BD group. Meanwhile, the relative abundance of *Lentisphaerae* in the CK, BR, and BD groups displayed a difference (*p* < 0.05), while the CK group was lower than the BR (*p* = 0.052) and BD (*p* = 0.054) groups. The relative abundance of *Tenericutes* in the BD group was higher than that in the CK and BR groups (*p* < 0.05).

**Figure 5:**
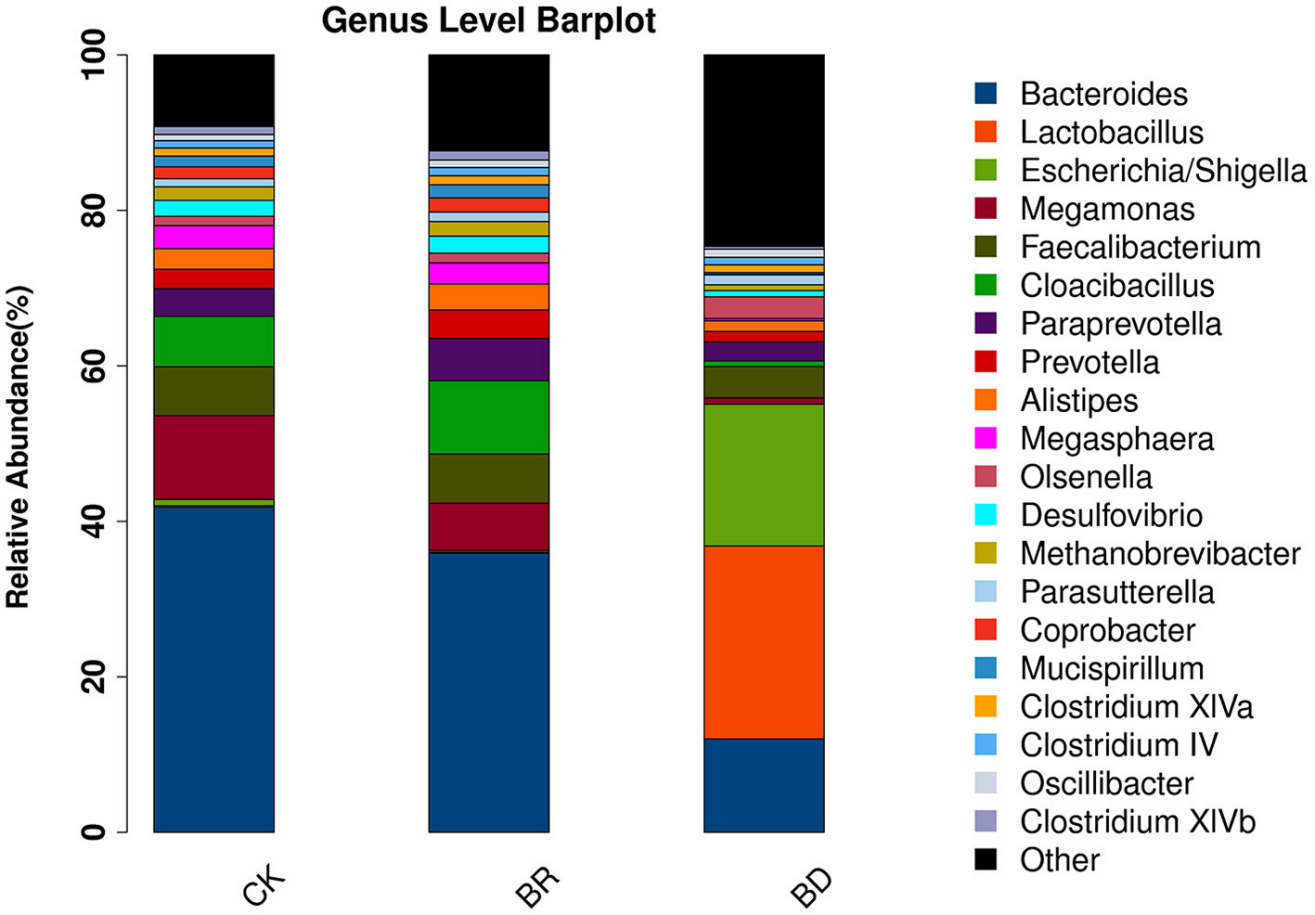
The different gut microbiota in the genus level. CK, check, *n* = 10; BR, broiler recipient, *n* = 10; BD, broiler donor, a pooled sample of *N* = 1.

At the top 20 of the genus level (Figure 5), the relative abundance of *Bacteroides, Megamonas, Cloacibacillus, Faecalibacterium*, and *Paraprevotella* were the top 5 microbiomes between the CK and BR groups. The relative abundance of *Bacteroides, Megamonas*, and *Faecalibacterium* in the BR group was 22.11, 46.83, and 5.20% lower than that in the CK group, respectively. The relative abundance of *Cloacibacillus* and *Paraprevotella* in the BR group was 26.84 and 33.53% higher than that in the CK group, respectively. Besides, the relative abundance of *Prevotella* was 35.54% higher and *Lactobacillus* was 47.95% lower in the BR group than those in the CK group. The relative abundance of *Lactobacillus* and *Escherichia/Shigella* in the quantity of data of the BD group was greater than that in the CK and BR groups (*p* > 0.05). *Megamonas* in the CK group was higher than in the BR (*p* = 0.06) and BD (*p* < 0.05) groups.

#### Linear Discriminant Analysis Effect Size Analysis Statistics of the Kyoto Encyclopedia of Genes and Genomes Different Pathway

**Figure 6:**
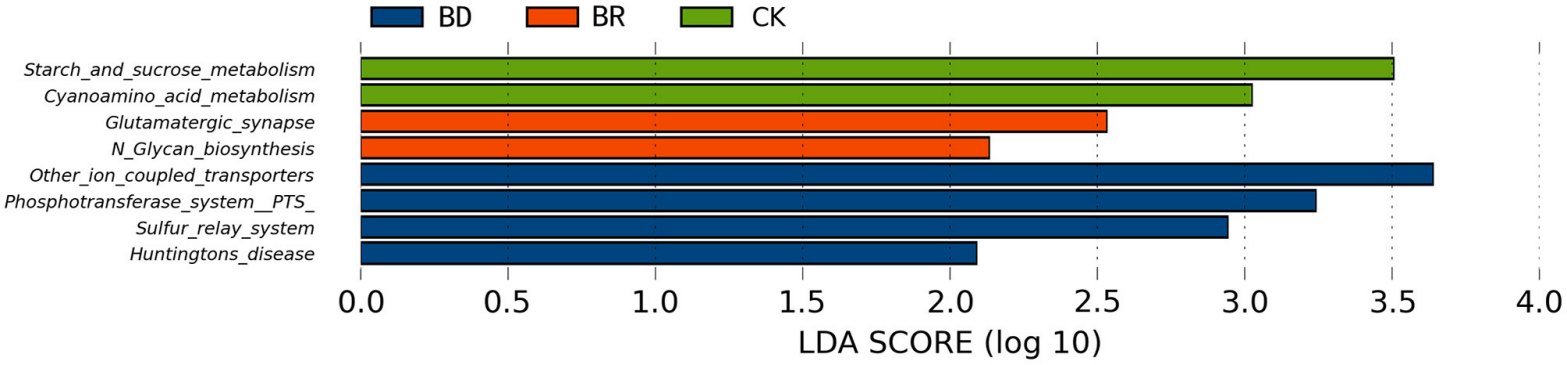
Linear discriminant analysis (LDA) effect size (LEfSe) analysis statistics of different Kyoto Encyclopedia of Genes and Genomes (KEGG) functional pathways among BD, BR, and CK groups. CK, check, *n* = 10; BR, broiler recipient, *n* = 10; BD, broiler donor, a pooled sample of *N* = 1. The different colors of the histogram represent different groups. The length of the histogram represents the LDA score, which represents the influence of significantly different pathways between groups. The bars listed are those that were LDA scores (log10) >2 and significantly different (*p* < 0.05) than the other groups.

Concerning the functional abilities of microbial communities, compared with BR (*n* = 10) and BD (a pooled sample of *N* = 1) groups, starch and sucrose metabolism (LDA score = 3.51) and cyanoamino acid metabolism (LDA score = 3.02) were enriched in the CK group (*n* = 10) (*p* < 0.05, Figure 6). Compared with the CK and BD groups, glutamatergic synapse (LDA score = 2.53) and N-glycan biosynthesis (LDA score = 2.13) were increased in the BR group (*p* < 0.05, Figure 6). Compared with the CK and BR groups, in the BD group, other ion-coupled transporters (LDA score = 3.64), phosphotransferase system (LDA score = 3.24), sulfur relay system (LDA score = 2.94), and Huntington's disease (LDA score = 2.09) were enriched (*p* < 0.05, Figure 6).

## Discussion

In recent years, FMT, serving as critical progress of the method in the medical field (10), has been widely applied in human and non-human studies and continues contributing its effect to the application on the production performance of farm animals. This study investigated the donor bacterial community in chickens and evaluated whether the application of FMT in the post-hatching period would modify cecal microbiota, promote body weight, and alter the small intestine (jejunum) histological morphology, and even behavior.

The donor characteristic in this study was similar to a probiotic-like product (1). Similar to a probiotic, FMT introduces microbiota into a recipient (40). In contrast to a probiotic, the microbial composition of FMT is a complex and diverse ecosystem (40). That is, FMT donors indicated that there was a better combination of intestinal microbiota in the makeup of this nature-made probiotic, which was more conducive to the reconstruction of recipient intestinal microbiota by introducing a complete and stable intestinal microbiota, even microbial metabolites (40). Therefore, FMT can be considered as a comprehensive and mixed probiotic to achieve the regulation and reconstruction of intestinal microbiota (41). Although the beneficial donor characteristic was finitely transferred to the receipt, the potential of the FMT in a probiotic-like administration is worth exploring and developing. *Lactobacillus* is one of the probiotics, whose quantity of data in the BD group was more than that of the CK and BR groups, relating to the advantages of gut microbiota to the colonization of gut microbiota for broilers. Similarly, abundant *Escherichia/Shigella* abundance is associated with high-feed efficiency in chickens (22), which is increased in the BD group. Post-hatching is a critical period of colonization of gut microbiota, during which the microbial structure is unstable and susceptible to external factors (1). From external microbial inoculum within the first days of life, it exerted some effects on manipulating gut microbiota. It is worth noting that the quantity of bacteria changed, and some bacteria losses occurred due to the FMT preparation steps.

The Venn diagram and the beta diversity (PCoA) showed a difference in line with a previous study, implying the changes in microbial structure induced by FMT (22). However, this study did not observe any change for alpha diversity regardless of a previous study that reported the decrease in Shannon and Simpson indexes after the treatment of FMT from 16 to 29 days post-hatching (22). That is to say, in this study, it is the microbial structure that is changed after FMT rather than the microbial richness. The reason may be attributed to the donor quality or the chicken breed, which needs to be further studied. In particular, it is critical to explore and illustrate the relationship of microbiota between BD and BR groups. It is conceivable that FMT did not introduce all bacteria from donor to recipient birds in accordance with a similar study (22), implicating a potential mismatch between the BD and BR groups. It is demonstrated that the CK and BR groups shared 729 common OTUs, and the BD group shared 531 and 542 OTUs with the BR and CK groups, respectively. Also, although the top three microbiomes at the phylum level were similar, the top five microbiomes at the genus level varied from one to another. Owing to transferring microbiota from the BD birds, the relative abundance of *Cloacibacillus, Paraprevotella*, and *Prevotella* were increased, while the relative abundance of *Bacteroides, Megamonas*, and *Lactobacillus* were decreased 20% more in the BR group compared with the CK group, respectively. It seems that these results showed no regular changes. The current understanding of the relationships between the BD and BR groups is still rudimentary. Therefore, it is difficult to draw a conclusion about the law of the FMT from donor to recipient birds in relation to transplanting microbiota in this study. The field remains in its infancy (41), and follow-up research is needed to gain a detailed understanding of the mechanisms for FMT. Nonetheless, the FMT application had a certain effect on intervening and shaping a microbial community and far-reaching effects on physiology in recipient birds (20–24).

A previous study reported that the lower relative abundance *Lentisphaera* and the higher *Megamonas* were found in high feed conversion ratio (FCR) birds compared with low FCR birds (42). *Lentisphaerae*, as one of the small-number phyla, plays a vital role in a healthy adult gut ecosystem, and *Megamonas* is associated with the improvement of the recovery of energy from food (43). In this study, there was a lower relative abundance of phylum *Lentisphaerae* and higher genus *Megamonas* in the CK group, suggesting that FMT exerts a limited effect of improving production performance in broilers. Importantly, the balance of the gut microbiota is critical for the chickens to exert an optimum function, including nutrient provision, competitive exclusion to pathogens, development of host immune system, and influence of intestinal morphological structure (1). As known, the gut microbiota is associated with production performance, such as improving FCR, increasing body weight, and so on (2). In this study, the current microbiota profile of the donor did not improve the body weight of broilers. This is inconsistent with the FMT increasing feed intake, which led to a gain of weight in female broilers (22). In addition, an important study indicates that obese and lean people have different gut microbial ecology (44), and transplanting the gut microbiota from obese people into germ-free recipients increases their body weight (45). Meanwhile, the microbial differences between CK and BR are limited. We speculated that the reasons may be attributed to the donor quality (a “favorable” microbiota, microbial composition, and even microbial metabolites) or breeds, which should be further optimized.

Developing a healthy intestine in birds plays a critical role in maintaining normal intestinal function. The muscle layer thickness and serous membrane layer thickness in the BR group were higher compared with the CK group, which may be supported by FMT could reduce epithelial injury (14) and alleviate the intestinal barrier injury (16). In addition, FMT from diseased bovine intestinal microbiota to mouse induced murine inflammation, including jejunum and colon tissue (19). These evidences demonstrate that exogenous microbiota exerts a critical role on intestinal morphology. The gut microbiota is essential to intestinal-barrier function regulation and modulates gut motility and intestinal barrier homeostasis so as to influence host physiology. In this study, intestinal morphology was improved by fecal microbiota suspension, to some extent, with thicker muscle layer thickness, and serous membrane layer thickness in the BR compared with the CK birds. Unexpectedly, the normal muscularis mucosa can promote gland secretion, which becomes thicker in the recipients. It is suspected to be related to the negative impacts of FMT, which should be optimized and eliminated.

Behaviors were found to be linked with the gut microbial composition; however, the causative mechanism remains unknown. For example, anxiety-like behavior is reduced in germ-free mice compared with specific pathogen-free mice (46). Chickens with high and low feather-pecking behavior display differences in gut microbiota (47, 48), and the feather-pecking behavior can be regulated and reduced by feeding probiotics *Bacillus subtilis* (49). In particular, post-hatching FMT did have effects on behavioral responses and physiological characteristics related to feather pecking (24). In quails, the fear behavior was divergent between germ-free and colonized individuals (50), and could be reduced via feeding probiotics (27). Similarly, compared with a high emotional line, the cecal microbiota transplantation with a low emotional line can influence emotional reactivity in Japanese quails (28). The vigilance and foraging in the novel arena tests and “response to predator” in the predator tests were higher in the CK group compared with those in the BR group, indicating higher fearfulness in the CK group. Besides, the BR group was increased in the glutamatergic synapse and N-glycan biosynthesis. There is emerging, but incomplete, evidence that enteric neurons influence the transport of glucose across the mucosa of the small intestine (51). The microbiota is increasingly recognized for its ability to influence the development and function of the nervous system and several complex host behaviors (52). The modulatory role of gut microbiota may boil down to the microbiome–gut–brain axis to influence the behavior (53, 54). In this study, exogenously administered FMT was found to affect gut microbiota functions and fear behavior. For the CK group, starch and sucrose metabolism and cyanoamino acid metabolism were enriched. Accumulating evidence demonstrates that gut microbiota participates in metabolic pathways, including carbohydrate metabolism, lipid metabolism, and amino acid metabolism (55). The different metabolism may respond to a higher relative abundance of *Megamonas* associated with improving the recovery of energy from food (42). Feces also harbors additional substances (proteins, bile acids, and vitamins), which might contribute to the recovery of gut function (41). For the BD group, Huntington's disease is predicted and enriched, which is a neurodegenerative disease in humans. It may be related to a mutation in Huntington's gene in the BD group. This result is unexpected, which may be attributed to the broiler in this study is a little slower, such as less active behaviors (56), less exploration, and more standing behavior (57). In addition, the KEGG database that is based on human KEGG pathways was used to predict microbial function, which is not necessarily matched for the functional pathways of chickens. However, metagenome analyses are capable of detecting microbial functions more accurately. All in all, FMT is able to introduce, establish, and alter the gut microbiota of the recipients, especially modulating crucial host functions (58).

FMT as a promising approach has a potential application in the production of animals associated with gut microbiota. The potential FMT mechanism might be the repair, replacement, and reconstruction of the primary microbiota of hosts by the healthy fecal microbiota and metabolites (12). Up to now, many areas concerning this emerging technology remain uncertain, such as the origin of a donor, the quality evaluation of a donor characteristic, the transmission of unknown organisms, and the monitor quantity of FMT, and so on. The FMT is not a state-of-the-art method, and the standardization of FMT is expected to be well established from human beings and non-human beings in the coming years (59).

## Conclusion

Some characteristics of the gut microbiota in the donor chickens could be transferred to the recipient chickens by fecal microbiota suspension. Exogenous fecal microbiota transplantation showed no difference in body weight, but improved fearfulness as well as muscle layer thickness and serous membrane layer thickness. Fecal microbiota transplantation as a probiotic-like administration could possibly be an efficient way to improve the physiological plasticity and behavior of chickens. Therefore, fecal microbiota transplantation could serve as a promising skill to improve animal health and welfare. Notably, the role of microbiota for various individuals and periods remains a secret, and the mechanism of microbiota on behaviors still awaits further investigation.

## Data Availability Statement

The datasets presented in this study can be found in online repositories. The names of the repository/repositories and accession number(s) can be found below: https://www.ncbi.nlm.nih.gov/, PRJNA725556.

## Ethics Statement

The animal study was reviewed and approved by China Agricultural University Laboratory Animal Welfare and Animal Experimental Ethical Inspection Committee (approval number: CAU20180628-6).

## Author Contributions

XBZ and SC obtained the funding. CY, SC, and XBZ designed this project. CY, SC, ZL, HL, XJZ, and JL performed the experiment. CY, XJZ, and SC analyzed and interpreted the data. CY, SC, and XBZ drafted and revised the manuscript. All authors agreed to the publication.

## Funding

This study received funding from the Joint Projects of Guizhou Nayong Professor Workstation, grant no: 201705510410352, the Joint fund of basic and applied basic research fund of Guangdong Province, grant no: 2019A1515110598, the Guangdong Provincial Key Laboratory of Animal Molecular Design and Precise Breeding, grant no: 2019B030301010, and the Key Laboratory of Animal Molecular Design and Precise Breeding of Guangdong Higher Education Institutes, grant no: 2019KSYS011.

## Conflict of Interest

The authors declare that the research was conducted in the absence of any commercial or financial relationships that could be construed as a potential conflict of interest.

## Publisher's Note

All claims expressed in this article are solely those of the authors and do not necessarily represent those of their affiliated organizations, or those of the publisher, the editors and the reviewers. Any product that may be evaluated in this article, or claim that may be made by its manufacturer, is not guaranteed or endorsed by the publisher.
